# Assembly and reasoning over semantic mappings at scale for biomedical data integration

**DOI:** 10.1093/bioinformatics/btaf542

**Published:** 2025-09-29

**Authors:** Charles Tapley Hoyt, Klas Karis, Benjamin M Gyori

**Affiliations:** Institute of Inorganic Chemistry, RWTH Aachen University, Aachen, NRW, 52074, Germany; Khoury College of Computer Sciences, Northeastern University, Boston, MA, 02115, United States; Khoury College of Computer Sciences, Northeastern University, Boston, MA, 02115, United States; Khoury College of Computer Sciences, Northeastern University, Boston, MA, 02115, United States; Department of Bioengineering, Northeastern University, Boston, MA, 02115, United States

## Abstract

**Motivation:**

Hundreds of resources assign identifiers to biomedical concepts including genes, small molecules, biological processes, diseases, and cell types. Often, these resources overlap by assigning identifiers to the same or related concepts. This creates a data interoperability bottleneck, as integrating data sets and knowledge bases that use identifiers for the same concepts from different resources requires such identifiers to be mapped to each other. However, available mappings are incomplete and fragmented across individual resources, motivating their large-scale integration.

**Results:**

We developed SeMRA, a software tool that integrates mappings from multiple sources into a graph data structure. Using graph algorithms, it infers missing mappings implied by available ones while keeping track of provenance and confidence. This allows connecting identifier spaces between which direct mapping was previously not possible. SeMRA implements a customizable workflow that takes a declarative specification as input describing sources to integrate with additional configuration parameters. We used SeMRA to produce the SeMRA Raw Mappings Database, an aggregation of 43.4 million mappings from 127 sources that jointly cover identifiers from 445 ontologies and databases. We also describe benchmarks on specific use cases such as integrating mappings between resources cataloging diseases and cell types.

**Availability and implementation:**

The code is available under the MIT license at https://github.com/biopragmatics/semra. The SeMRA Raw Mappings Database assembled by SeMRA is available at https://doi.org/10.5281/zenodo.11082038.

## 1 Introduction

Consistent and standardized identification of biomedical entities is an important step in creating and maintaining FAIR (Findable, Accessible, Interoperable, and Reusable) (Wilkinson *et al.* 2016) data. However, many ontologies and databases that catalog biomedical entities are partially overlapping. For instance, cancer cell lines appear in several ontologies (Malone *et al.* 2010, Bairoch 2018, Gremse *et al.* 2011, Sarntivijai *et al.* 2014), databases (Romano *et al.* 2009, Barretina *et al.* 2012, Vempati *et al.* 2014, Ghandi *et al.* 2019), and generic nomenclature resources (Rogers 1963, Bodenreider 2004, Noy *et al.* 2008). As a specific example, the human astrocytoma cancer cell line 13210N1 appears in Cellosaurus (Bairoch 2018) under the identifier cellosaurus:0110 and in the Cell Line Ontology (CLO) (Sarntivijai *et al.* 2014) as CLO:0001072.

Recognizing equivalent entries in different databases and mapping or merging across these is crucial for data interoperability and data analysis spanning multiple sources. It is also critical for computational tasks such as the construction of knowledge graphs (Himmelstein *et al.* 2017, Nicholson and Greene 2020, Friedrichs 2021), automated systems biology model assembly (Gyori *et al.* 2017, Bachman *et al.* 2023), finding identifiers for bioentities mentioned in text (Gyori *et al.* 2022), and integrating ontologies (Lambrix and Tan 2008, Geleta *et al.* 2022, Guo *et al.* 2022). Each of these tasks requires mappings (also called *ontology mappings* or *semantic mappings*) across two or more resources that specify *equivalence* or other relations such as *broad*, *narrow*, or *related* between specific entries of the two resources. Mappings also ideally provide metadata on provenance and confidence to allow usage in a principled way. We refer to Figure 2 of Matentzoglu *et al.* (2022a) for a more detailed description of mappings. Overall, semantic mappings are crucial for consistent integration across resources.

### 1.1 Problem statement

Semantic mappings are often made available by individual (“primary”) resources, for example, most Open Biological and Biomedical Ontologies (OBO) (Jackson *et al.* 2021) provide cross-references to equivalent or related entries in overlapping ontologies. In addition, “secondary” mapping resources including aggregators such as BridgeDB (van Iersel *et al.* 2010), TogoID (Ikeda *et al.* 2022), or independent mapping repositories like Biomappings (Hoyt *et al.* 2023) provide mappings that are collected from multiple sources or extend upon what is available from individual resources using additional curation. However, mappings remain difficult to assemble at scale because of the variety of *ad hoc* storage formats they are made available in, the ways they are produced (e.g. manual curation, rule-based inference, lexical matching), and the availability of metadata (e.g. precise mapping relations, curator confidence). A survey from Laadhar *et al.* (2020) of mappings in life science ontologies highlighted several widespread issues such as the use of unspecific predicates (e.g. oboInOwl:hasDbXRef, which is widely used to represent cross-reference mappings but is not specific enough to assert exact equivalence) and a lack of detailed provenance metadata. The Simple Standard for Sharing Ontological Mappings (SSSOM) (Matentzoglu *et al.* 2022a) was developed to support curation of more specific predicates and detailed provenance metadata and is gaining adoption across many resources as a standard; however, it remains the case that most available mappings do not follow a standard format or semantics.

**Figure 1.**
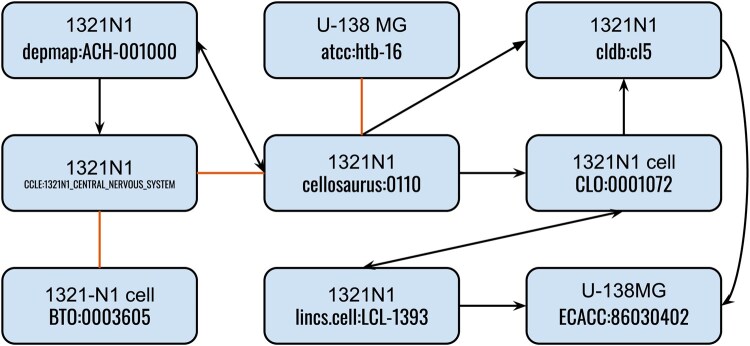
1321N1 is a cancer cell line derived from an astrocytoma of a 47-year-old male Caucasian patient. It is characterized by prominent pathogenic single nucleotide variants in the PTEN (HGNC:9588) and TP53 (HGNC:11998) genes. It appears in a variety of cell line resources (e.g. Cellosaurus), anatomy resources (e.g. BTO), clinical databases (e.g. DepMap, CCLE), and vendor catalogs (e.g. Sigma Aldrich). Solid black lines denote primary mappings pointing away from the resource from which the mapping was derived. Orange lines denote secondary mappings from Biomappings (Hoyt *et al.* 2023).

Even if one were to assemble primary and secondary mappings at scale, there often exist gaps. Figure 1 demonstrates that the landscape of mappings between cancer cell line resources is highly fragmented, and inference over multiple mappings is often necessary to identify key equivalences, e.g. between the BRENDA Tissue Ontology (BTO) (Gremse *et al.* 2011) and CLO entities. The Ontology Mapping Service (OXO) (Harrow *et al.* 2019) imports low-precision database cross-references from biomedical ontologies and implements inference across these, but is susceptible to data quality issues, explosions due to many-to-many mappings, and excludes valuable nomenclatures [e.g. the NCBI Entrez Gene Database (Maglott *et al.* 2011)] that are not curated as ontologies. Notably, the landscape of mappings between resources is highly scattered, such that a combination of several mappings is required to identify equivalences, e.g., between the BTO entity and the CLO entity in Fig. 1.

There are several other problems that currently limit the use of mappings for data integration. First, though standards like SSSOM have recently been developed to represent simple provenance for mappings, there does not yet exist a provenance model that can capture provenance of mappings inferred from one or more other mappings. Second, there do not yet exist methods that incorporate uncertainty associated with the provenance of a mapping into measures of confidence for inferred mappings. Uncertainty results from, for example, noisy mapping algorithms (e.g. lexical prediction), implicit one-to-many mappings due to broad or narrow matches, and curation error. An appropriate confidence model would incorporate both assumptions about how a mapping is produced and its intended interpretation for a specific data integration task. Third, there does not yet exist a data model or workflow to declare implicit prior knowledge about the interpretation of cross-references from specific sources. For example, it is known that the mappings distributed in an *ad hoc* TSV file from the Cancer Dependency Map Project (Barretina *et al.* 2012) to Cancer Cell Line Encyclopedia (CCLE) (Ghandi *et al.* 2019) are *equivalences*, while only a subset of the low-precision mappings in Cellosaurus to CLO represent equivalences.

### 1.2 Contribution

To address these issues and open problems, we introduce the Semantic Mapping Reasoning Assembler (SeMRA), a novel method for automatically assembling mappings at scale, implemented as configurable open-source software. SeMRA represents mappings as a directed graph and provides functionality to infer indirect mappings based on graph traversal, then determine associated confidence. Importantly, it allows for customizing prioritization order to merge equivalent concepts during data integration in a consistent way. SeMRA further implements graph-based algorithms for flagging mappings that lead to inconsistent data integration as part of a semi-automated quality assurance workflow. It implements a locally deployable web application for the interactive exploration of mappings, visualization of mapping graphs, and curation of flagged mappings.

We used SeMRA to assemble 43.4 million raw semantic mappings from 127 sources (including widely used biomedical ontologies and databases) to make available the broadest-coverage integrated mapping resource to date. In addition, we provide detailed analysis and metrics of using SeMRA on specific areas of biomedicine crucial for data integration. First, we demonstrate integrating cell and cell line resources using SeMRA, significantly expanding on results from Hoyt *et al.* (2023). Second, we integrate resources cataloging diseases and show substantial expansion in scope compared to the results published by Haendel *et al.* (2020). Finally, we provide metrics on the effect of semantic integration using SeMRA on overlaps between resources in five areas of biomedicine.

SeMRA is available as an open-source Python package (https://github.com/biopragmatics/semra) that can be installed with pip install semra. We include code examples in the Supplementary Material, available as supplementary data at *Bioinformatics* online and full documentation on ReadTheDocs at https://semra.readthedocs.io. We also provide the SeMRA Raw Mappings Database, a comprehensive mapping database in the SSSOM exchange format archived on Zenodo at https://zenodo.org/records/15208251.

## 2 Materials and methods

**Figure 2.**
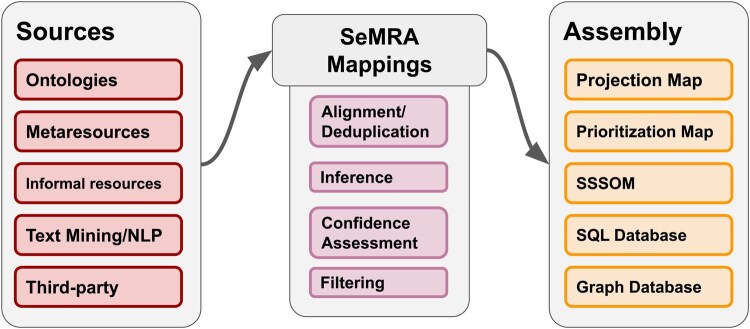
An overview of the software architecture of the SeMRA Python package. Source modules provide ingestion logic from various sources, feeding into a set of central SeMRA Mappings modules that compile mappings serving as input to Assembly modules that produce outputs in multiple export formats.

SeMRA is built with a modular architecture (Figure 2) with independent components for processing input sources, a semantic mapping data structure, inference and processing, assembly, export into multiple file formats, export to a graph database with a JSON API, and a locally deployable interactive web application.

### 2.1 Sources

SeMRA implements a variety of import modules and processors for mappings. Where possible, SeMRA wraps preexisting parsers for standard representations. For instance, SeMRA reads mappings from ontologies in OBO format by wrapping the PyOBO Python package (Hoyt *et al.* 2025). Similarly, SeMRA reads mappings from ontologies in the OWL and OBO Graph JSON formats using the Bioontologies Python package (Hoyt and Gyori 2025). However, there are a number of ontologies that require custom processing due to nonstandard encoding of mappings, such as the CLO (Sarntivijai *et al.* 2014). These are implemented in SeMRA with additional custom logic.

SeMRA also reads mappings in the SSSOM format via the sssom-py Python package (Hegde *et al.* 2025). This also gives access to several other formats, including the Expressive and Declarative Ontology Alignment Language (EDOAL; David *et al.* 2011) and the RDF Alignment Format (Anguita *et al.* 2013), both used by the Ontology Alignment Initiative. Notably, support for SSSOM allows SeMRA to import mappings from Biomappings (Hoyt *et al.* 2023), a resource dedicated to predicting and curating mappings missing from primary sources.

SeMRA integrates with Wikidata (Vrandečić and Krötzsch 2014) in order to automatically generate SPARQL queries for properties corresponding to various ontologies and databases (e.g. P2158 for the CLO).

More generally, SeMRA implements a plug-and-play concept to implement custom parsers and importers for sources that are not in a standard format. Consequently, we implemented custom parsers to import mappings from OMIM, IntAct, PubChem, NCIT, ChEMBL, and several other resources. Similarly, SeMRA can be extended by users to process additional resources that are not available in a standard form.

### 2.2 Semantic mapping data structure

SeMRA implements a hierarchical data structure to represent mappings. The core data structure represents each unique mapping triple with a *subject*, *predicate*, and *object*. The *subject* and *object* represent entities in ontologies or databases using the compact universal resource identifier (CURIE) syntax (Birbeck and McCarron 2010). CURIEs are automatically validated against the Bioregistry standard (Hoyt *et al.* 2022) to ensure consistent usage. The *predicate* comes from Simple Knowledge Organization System (SKOS) or related vocabularies (see https://mapping-commons.github.io/sssom/mapping-predicates/).

Each core triple has one or more evidence objects associated with it. There are two types of evidence objects. A *simple* evidence object contains a mapping justification based on the Semantic Mapping Vocabulary (SEMAPV) (Matentzoglu *et al.* 2022b), an optional author annotation using an ORCID identifier, an optional confidence annotation from the producer, and a reference to the mapping set from which it came. Each mapping set has a name, version, license, and consumer confidence annotation. A *complex* evidence object represents the results of reasoning and contains pointers to all mappings used to construct the evidence, a mapping justification from SEMAPV, and an optional confidence factor reflecting the consumer’s confidence in the reasoning approach.

SeMRA’s mapping data structure is best suited for sequential operations. For other operations, including inference (see Section 2.3), a directed graph data structure is more appropriate. Each SeMRA edge can be compiled into a graph by making the subject of the mapping the source node, the object of the mapping the target node, and the predicate (and related evidence) properties on the edge. Mapping triples are inherently directional, despite many predicates being symmetric. Therefore, SeMRA explicitly models the directionality of each predicate while enabling inference of symmetric mappings in order to explicitly track provenance of operations.

*Comparison to SSSOM*. SeMRA’s data model captures all required and commonly used optional fields in SSSOM. However, a conceptual distinction is that SSSOM represents individual mapping triples with a single supporting evidence as part of the mapping data structure, whereas SeMRA decomposes the representation of triples and the associated evidence. This allows for integration of evidence from multiple sources and a more sophisticated inference logic.

*Confidence*. Confidence associated with a mapping is calculated in a hierarchical manner based on the set of evidence supporting it, inspired by Bachman *et al.* (2023). We interpret the confidence of a specific evidence for a mapping as a probability value between 0 and 1, and denote it $c_{e}$ for a given evidence *e*.

To characterize $c_{e}$, in case of a *simple* evidence, SeMRA takes into account both the producer’s confidence (if any is provided) for its mappings $c_{\text{Producer}}$ and the confidence of the consumer (in this case, SeMRA or its user) in the mapping set the evidence came from: $c_{\text{MappingSet}}$. The evidence confidence is then expressed as $c_{e}=1-(1-c_{\text{MappingSet}})(1-c_{\text{Producer}})$. If no producer confidence is given, it is assumed to be 1.0, therefore simplifying the formula to directly using the consumer’s confidence in the mapping set. Assuming a mapping is supported by a set of evidences *E*, its overall confidence is expressed as the probability that at least one supporting evidence is correct: $c_{m}=1-\prod_{e\in E}1-c_{e}$ for mapping *m*.

Finally, the confidence for a *complex* evidence is calculated from the confidence of the mappings it is derived from: $c_{e}=\gamma_{op}(1-\prod_{m\in M}\left(1-c_{m}\right))$ where $\gamma_{op}$ is a scaling factor specific to reasoning operation *op* used.

*Identifying mappings through hashing*. Several applications require the unique identification of mappings and evidences associated with mappings. However, this is difficult because each data structure composes multiple entities. In SeMRA’s context, a mapping’s identifier has to reflect the content of the mapping it represents to avoid inconsistencies in the highly dynamic and decentralized nature of the creation of mappings and evidences. SeMRA therefore implements a deterministic method for hashing mappings, evidences, and mapping sets independently that can be used for unique identification of each resource within the SeMRA ecosystem.

### 2.3 Inference and processing

**Figure 3.**
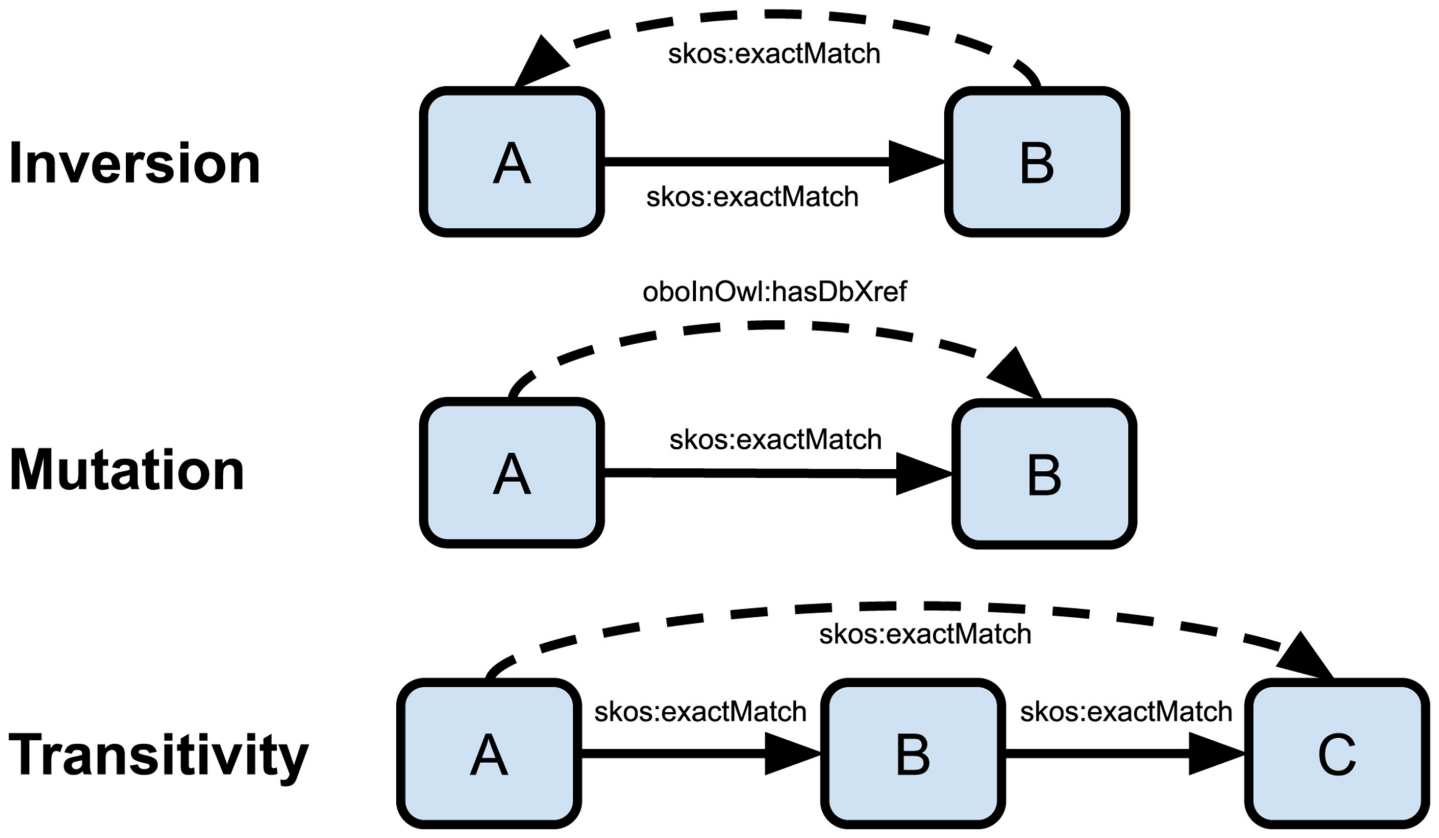
An overview of inference methods implemented in SeMRA, nodes representing entities between which edges represent mappings. The edge labels show examples of predicates involved in the inference steps with solid lines representing original edges and dashed lines representing inferred edges.

SeMRA implements three inference methods: inversion, mutation, and transitivity. Using these inference methods, new mappings can be inferred from existing mappings or existing mappings can be made more specific, thereby broadening downstream data integration capabilities. These inference methods implemented in SeMRA are consistent with chaining rules described in the SSSOM documentation (Fig. 3) (https://mapping-commons.github.io/sssom/chaining-rules) to ensure consistency with community expectations of inferred mappings. These functionalities are exposed along with several filtering approaches through a high-level function called semra.pipeline.process(). We next describe each inference method in detail.

*Inversion*. Inversion creates a new mapping where the subject and object are swapped and the predicate is changed. For symmetric predicates, such as those representing equivalence, the same predicate is reused. For example, if A is an exact match of B, then B is also an exact match of A. For asymmetric predicates, a correspondence between inverse predicates is maintained to facilitate inversion. For example, if A is a narrow match of B, then B is a broader match of A. In a directed mapping graph, this is equivalent to adding a reverse edge.

*Mutation*. Mutation creates a new mapping in which the original predicate is replaced by a new one. Mutations that generalize a stronger predicate with a weaker one are pre-configured in SeMRA. This is useful when integrating sources using a combination of strong OWL-based semantics and weaker SKOS-based semantics. For example, if A is an OWL equivalent class to B, then A is also a (SKOS) exact match to B. In a directed mapping graph, this is equivalent to adding a parallel edge.

Mutations that ascribe stronger semantics to a weaker predicate are also useful, but are often context- or task-specific. For example, some modeling approaches do not distinguish between genes and proteins, and therefore use their identifiers interchangeably. In this scenario, the “has gene product” (RO:0002205) relation between genes and proteins can be safely mutated to an exact match predicate. Similarly, many OBO Foundry ontologies store implicitly exact matches in the database cross-reference field, and therefore lose their precision. SeMRA allows the prior knowledge about each ontology’s curation practices to be explicitly configured during inference.

*Transitivity*. Transitivity rules allow new mappings to be inferred from chains of two or more mappings. For example, if A is an exact match to B and B is an exact match to C, then A is an exact match to C. Transitivity rules can also incorporate more complex chaining rules with different predicate types. Inference based on transitivity rules is implemented in SeMRA using the directed mapping graph data structure. With this, it implements a custom graph traversal related to breadth-first search that incorporates transitivity rules, resolution of conflicting mappings, and filtering of negative mappings. In a directed mapping graph, this is equivalent to adding an edge corresponding to a path through the graph.

*Filtering*. SeMRA implements several filtering operations, which are often useful to automatically identify and remove false-positive mappings generated by inference. First, filtering mappings based on their source prefix, target prefix, or source-target prefix pair is often useful to remove irrelevant mappings for a given context. Similarly, some pairs of source-target prefixes can be excluded from prior knowledge. For example, terms in disease-related ontologies like MONDO (Vasilevsky *et al.* 2025) should never be mapped to terms in gene-related resources like the NCBI Entrez Gene Database (Maglott *et al.* 2011). Second, filtering by cardinality includes the identification of entities in one resource that have exact matches to more than one entity in another resource. These do not allow consistent data integration and present an opportunity for additional curation. Third, filtering by confidence presents another automated method for removing potentially low-confidence mappings produced by a combination of low-confidence steps, such as mutation.

### 2.4 Assembly

SeMRA provides several high-level workflows for assembling mapping sets into useful artifacts, several input and output functions for reading and writing mappings, and several export modes for building mapping databases.

*Projection mapping set*. A projection mapping set contains mappings that support mapping identifiers from one ontology or database to another. In a projection mapping set, all subjects’ prefixes are the same, and all objects’ prefixes are the same; however, they may contain a combination of simple and complex evidence arising from primary sources and inference approaches. Projection mappings help data integration between two specific resources. SeMRA implements a workflow for creating a projection map that can be stored in either SSSOM or a highly compressed tabular format.

*Prioritization mapping set*. A prioritization mapping set defines for each entity what the standard representation of that entity should be. Therefore, each entity appears as the subject in exactly one mapping, and some entities may appear as the object of several mappings. Effectively, a prioritization mapping induces star graphs for each clique of mutually equivalent entities. In order to create a prioritization mapping set, a prioritization list of prefixes is required. There is also an assumption that only one entity from each prefix is in each clique.

A prioritization mapping set is useful in knowledge integration tasks where multiple sources with overlapping identifiers (such as some of the resources shown in Fig. 1) are used, across which terms need to be standardized to a single prioritized identifier.

### 2.5 Export and exploration

SeMRA provides several export formats. First, it provides a low-level, direct export of its data using Python’s pickle module. Second, it provides an export to SSSOM, which enables conversion via sssom-py to several other formats (RDF, OWL), and provides an upload option to the NDEx platform (Pratt *et al.* 2015).

*Graph database*. SeMRA implements a workflow for serializing a mapping set into the Neo4j graph database (https://neo4j.com), which enables custom queries and can serve as a backend for user applications. The graph represents concepts, evidence, mappings, and mapping sets as nodes. It also represents mappings as edges between two concepts (representing mappings both as nodes and edges provides query flexibility), and further represents as edges the interdependencies between mapping sets, evidence, mappings, and concepts. SeMRA exports files for building the database along with a Docker configuration and startup scripts to enable any mapping set to be turned into a database.

*Web application*. SeMRA also implements a locally deployable web application on top of its graph database that exposes several high-level operations as a JSON API built with FastAPI. It implements a user interface containing a dashboard for summarizing the concepts, mappings, evidence, and mapping sets in the database as well as for exploring mappings, concepts, and cliques of equivalent entities (Fig. 1, available as supplementary data at *Bioinformatics* online). For specific questions, the graph database can be queried directly using the Cypher query language (see Supplementary Material, available as supplementary data at *Bioinformatics* online). The SeMRA web application has an optional integration with the Biomappings package (Hoyt *et al.* 2023) for the curation of negative mappings. It gives users direct insight into cliques that have problematic features such as containing multiple entities from the same resource that can be curated and then incorporated into future inference.

## 3 Results

We first present an overall integrated database of mappings using SeMRA. Then, to show SeMRA’s configurability and use case-specific utility, we provide several processing pipelines with a more specific scope, as follows. In Section 3.2, we integrate semantic mappings across 10 cell type and cell line resources. In Section 3.3, we apply SeMRA to integrating resources that catalog human diseases to expand on the analysis in Haendel *et al.* (2020). In Section 3.4, we quantify the effect of semantic integration with SeMRA on resolving redundancies in five areas of biomedicine (including cells and diseases from the previous two case studies).

### 3.1 Integrated mapping database

Using SeMRA, we generated the SeMRA Raw Mappings Database, a database of 43.4 million mappings from 127 sources, including ontologies, databases, and custom sources. Of these, 36.8 million were exact matches, 5.5 million were imprecise database cross-references, and around 1 million were other types of mappings. The mappings cover entities from 445 different ontologies and databases, due to the fact that a given source often maps to several external ontologies and databases. SeMRA imports life and natural science ontologies from the entirety of the OBO Foundry (Jackson *et al.* 2021), a high-quality subset of BioPortal (Whetzel *et al.* 2011), and other ontologies that have been curated in the Bioregistry (Hoyt *et al.* 2022) based on community-driven use cases. SeMRA also imports databases that are generally of interest in biomedical data integration through ontology adapters in the PyOBO software package or through custom importers. For example, this includes widely used chemical databases like ChEMBL (Gaulton *et al.* 2017) and gene databases such as NCBI Entrez Gene (Maglott *et al.* 2011).

The SeMRA Raw Mapping Database is available from Zenodo at https://zenodo.org/records/15208251 as SSSOM and as a pre-built graph database deployable via Neo4j. The SSSOM format explicitly states the license for each mapping, ultimately reflecting the mapping source’s license. It can be rebuilt using a single command semra build running in around 7 hours on commodity hardware.

This demonstrates the application of SeMRA at scale, to a general collection of widely used resources.

### 3.2 Landscape of cell and cell line resources

Resources cataloging cell types and specialized disease cell lines are a cornerstone of biology research and are crucial for the interpretation of single-cell data (Karlsson *et al.* 2021) and cancer gene dependencies (Tsherniak *et al.* 2017). Multiple resources exist providing entries for cell types and cell lines, including dedicated databases such as Cellosaurus and CCLE as well as resources with a broader scope such as the Experimental Factor Ontology (Malone *et al.* 2010), or UMLS (Bodenreider 2004). The major challenge remains that these databases overlap, but mappings between them are limited.

We used SeMRA with a declarative configuration as input to run semantic integration on ten relevant resources (Table 1, available as supplementary data at *Bioinformatics* online). In addition to primary mapping sources from each resource, SeMRA made use of the semi-automatically curated mappings described by Hoyt *et al.* (2023). SeMRA inferred thousands of new mappings that would not have been available without such integration. This included the production of the first mappings between several resources, including EFO-UMLS, CCLE-CLO, MeSH-Cellosaurus, and BTO-NCIT, which have thus far not been available from other sources.

Next, we used SeMRA to programmatically identify several systematic issues in cross-references from Cellosaurus, which often map to multiple terms in the CLO. We used a local deployment of the SeMRA web application to interactively review these issues, identify the primary mappings causing many-to-many references, and directly curate overrides in Biomappings. In several situations where database cross-references were to narrower or broader terms, we were able to curate negative mappings with the SeMRA web application’s Biomappings plugin. We found this was the case since there are often duplicate terms in CLO for the same cell line, imported from multiple external resources without normalization. This suggests that further work is necessary to address issues where a given resource has multiple terms for the same concept that should be mapped jointly. The resulting SeMRA Cell and Cell Line Mappings Database is available as a set of data files, and Dockerized graph database and a locally deployable web application at https://zenodo.org/records/15164183. It can be rebuilt using python -m semra.landscape.cell in around one hour using commodity hardware.

### 3.3 Landscape of disease resources

The characterization and classification of diseases have a rich history. Computational resources cataloging diseases enable the standardized annotation of health data from clinical trials, drug indications, electronic health records, and other artifacts. They further support computational efforts in drug discovery, target prioritization, and modeling. However, the number of tasks they support has led to a high proliferation of disease resources with high overlap.

We used SeMRA with a declarative configuration as input to run semantic integration on 16 relevant resources (Table 2, available as supplementary data at *Bioinformatics* online) including ontologies, databases, billing code systems, and generic resources. Our analysis showed that there is high redundancy between these resources that requires a union and deduplication via a combination of mappings from primary resources, secondary resources, and inference. While MONDO and UMLS have each been previously positioned as disease mapping hubs, our analysis suggests that they are by themselves insufficient, and that an automated mapping assembly pipeline can serve as a generalization of the purpose of MONDO as a mapping hub suggested by Haendel *et al.* (2020).

Unlike the cell line scenario, where the described resources could be directly downloaded, the disease resource scenario presents the problem that several resources (ICD10-CM, ICD9, ICD9-CM, and OMIM) cannot be directly downloaded either due to lack of availability or usage restrictions (in the case of OMIM). Therefore, this analysis is only able to estimate a lower bound for the number of terms appearing in these resources based on the ones appearing in mappings. The resulting SeMRA Disease Mappings Database is available as a set of data files, and Dockerized graph database and a locally deployable web application at https://zenodo.org/records/15164180. It can be rebuilt using python -m semra.landscape.disease in around 2 hours using commodity hardware.

### 3.4 Meta-landscape analysis

We performed landscape analyses over three additional domains to the first two case studies (anatomy, protein complexes, and genes) in order to estimate the total number of terms across common resources, as well as to use mappings to estimate the number of consolidated terms (Table 1). An example configuration is shown in Fig. 2, available as supplementary data at *Bioinformatics* online; configurations for each domain can be found at https://github.com/biopragmatics/semra/tree/main/src/semra/landscape.

We found that semantic mapping assembly resulted in a meaningful consolidation of redundant terms within each domain (reduction column in Table 1). Specifically, the high redundancy of resources in the domains of diseases and protein complexes leads to the largest reductions, especially given their relatively small size. Notably, the reduction for the gene domain is small because the number of model organism databases and the number of genes in each is only a small fraction of the NCBI Entrez Gene Database, which contains genes for several orders of magnitude more organisms. However, these estimations come with several caveats. First, these estimations can be artificially high because of missing mappings or due to the removal of many-to-many mappings during processing. Second, these estimations could be artificially low due to incorrect mappings or due to the missing terms from vocabularies that could not be accessed in full (e.g. SNOMED-CT for diseases).

Finally, we note that this analysis can be extended to other domains by writing new configurations for SeMRA. These could include other common entity types in biomedical research such as chemicals, protein families, pathways, molecular functions, assays, and taxa.

**Table 1.** Meta-landscape analysis statistics.^a^

Domain	Raw terms	Unique terms	Reduction	Zenodo ID
Disease	377 250	275 044	27.1%	11091886
Anatomy	39 362	33 877	13.9%	11091803
Protein complex	17 932	9011	49.7%	11091422
Gene	58 382 593	57 660 624	1.2%	11092013
Cell and cell line	218 557	172 299	21.2%	11091581

a Each Zenodo record contains all raw mappings, processed mappings, and a Dockerfile for locally running the graph database, JSON API, and SeMRA web application for the domain.

## 4 Discussion

We presented SeMRA, a configurable workflow for automatically assembling mappings at scale and an associated mapping database of 43.4 million mappings. SeMRA can be used to assemble context- and task-specific processed mappings using a combination of graph-based inference to infer indirect mappings, *a priori* domain knowledge, and custom prioritization that enables merging equivalent concepts.

The SeMRA Raw Mappings Database is broader in scope than existing work on semantic mapping extraction and integration. For example, Laadhar *et al.* (2020) extracted 1 million database cross-references from a subset of OBO Foundry comprising 30 ontologies in order to characterize the nature and quality of cross-references, but did not attempt to comprehensively cover the 100+ OBO Foundry ontologies, make inferences, detect inconsistencies, nor provide reusable software. The OXO imports most of the OBO Foundry and UMLS for supplementary mappings and contains 830 thousand mappings (as of 2024). TogoID and BridgeDB are relatively broader in coverage with 103 and 148 resources included, respectively. However, both BridgeDB and TogoID are limited in that they do not employ inference or make their results readily available in reusable formats.

We again highlight that the domain-specific semantic mapping assembly workflows enabled the consolidation of up to 49.7% of terms in a given domain. The artifacts from the disease, cell, and other landscape analyses enable data scientists to semi-automatically integrate data and knowledge from disparate resources at scale, and have higher confidence that duplicate concepts identified by different ontologies are consolidated, leading to better results of downstream analysis that leverages such integration work.

### 4.1 Limitations

SeMRA’s reasoning approaches are inherently limited by the availability of mappings from the set of sources we integrated and the quality of such mappings. To mitigate this, the modular nature of SeMRA allows additional sources to be readily added by curating new prefixes and their associated ontology artifacts in the Bioregistry (Hoyt *et al.* 2022), by implementing additional database plugins in PyOBO (Hoyt *et al.* 2025), or by directly implementing custom processors in SeMRA. However, more generally, the limitations of existing mapping sources highlight the need to incorporate semi-automated mapping curation workflows like Biomappings (Hoyt *et al.* 2023); automated approaches like Logmap (Jiménez-Ruiz and Cuenca Grau 2011), LOOM (Ghazvinian *et al.* 2009), and K-Boom (Mungall *et al.* 2019); and LLM-based automated review workflows like MapperGPT (Matentzoglu *et al.* 2023).

The integration of mappings across fragmented sources is also challenging due to the fact that primary sources use inconsistent semantics when distributing mappings (for instance, as we observed in the case study on cell lines, there are database cross-references that result in many-to-many mappings). SeMRA attempts to mitigate this issue by (i) making available a combination of mutation features, (ii) configurability for the custom prioritization of resources across which mappings are traversed, and (iii) automated checking for inconsistencies induced by integrated mappings. Still, correctly resolving such inconsistencies is highly challenging and, in some use cases, requires further custom logic beyond the starting point that SeMRA provides.

### 4.2 Future work on SeMRA and future vision

There are several open questions for developing methods for the inference and assembly of mappings. SeMRA does not yet explicitly handle alternative identifiers and replaced-by annotations in ontologies that describe when multiple terms cover the same concept. Further, splitting and merging terms creates additional challenges in reconciliation needed before making consistent mappings.

It would also be productive to explore how the construction of existing aggregate resources such as BridgeDB could be reproduced with a declarative configuration and automated pipeline using SeMRA. We expect this could significantly reduce the engineering effort needed to maintain such resources and increase the domains over which they are applicable.

We will also work toward improving the user experience for mapping curation in the SeMRA front-end in order to support and enable additional community curation. For example, this could be done by using SeMRA as a backend for feature-rich mapping curation interfaces like Cocoda (Ribeiro and Cerveira 2018). However, irrespective of the interface, custom curation will require architectural support in SeMRA for integrating these during the generation of its mapping resource output, which is done statically due to inference typically being a slow process necessitating.

SeMRA has the potential to be an important component of a broader ecosystem to actively monitor and support the maintenance of ontologies and databases. In this context, SeMRA could propose inferred mappings for curation by primary resource curators and alert them to inconsistencies (such as those caused by many-to-many mappings) on a timely basis. Such a service could be easily incorporated into resources that adhere to the open code, open data, open infrastructure (O3) guidelines (Hoyt and Gyori 2024), such as OBO Foundry ontologies. Ultimately, we envision SeMRA will act as an intermediate semantic interoperability layer allowing for the consistent usage of identifier resources in biomedicine and other fields.

## Supplementary Material

btaf542_Supplementary_Data
