# Supplementary material for: Assembly and reasoning over semantic mappings at scale for biomedical data integration
Source: Bioinformatics. 2025 Sep 29;41(11):btaf542. doi: 10.1093/bioinformatics/btaf542 (PMC12629235; doi:10.1093/bioinformatics/btaf542)
Supplement: btaf542_Supplementary_Data [file btaf542_supplementary_data.pdf]

Supplementary material: Assembly and reasoning over semantic mappings at scale for biomedical data integration

Web application

SeMRA provides a locally deployable web application to browse mappings, a snippet of which is shown in Supplementary Figure S1.

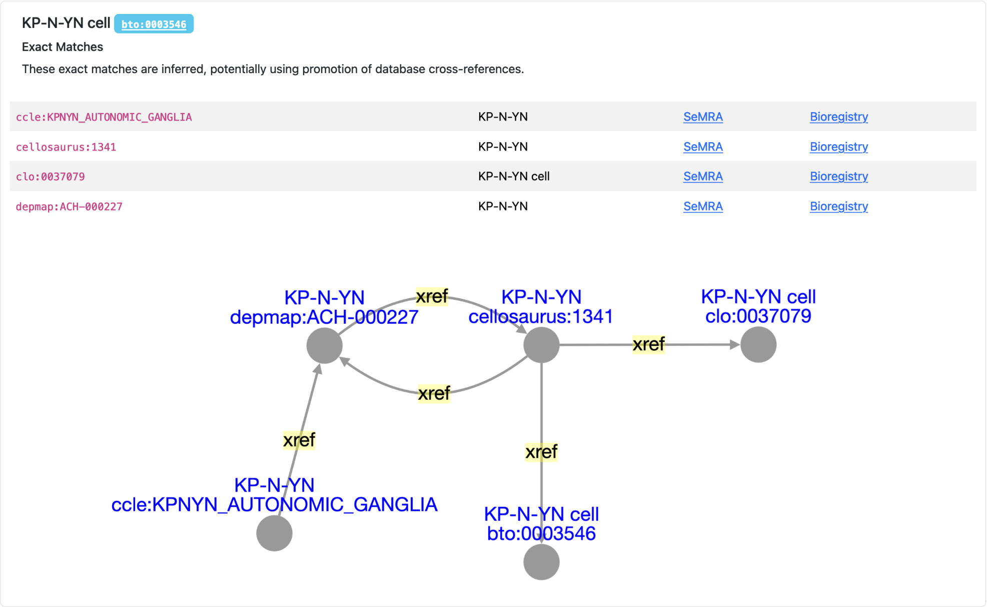

Supplementary Figure S1: A view over the locally deployable SeMRA web application.

Declarative configuration

```json
{
  "name": "Anatomy mappings",
  "inputs": [
    {"source": "biomappings"},
    {"source": "gilda"},
    {"source": "pyobo", "prefix": "uberon", "confidence": 0.99},
    {"source": "pyobo", "prefix": "bto", "confidence": 0.99},
    {"source": "pyobo", "prefix": "caro", "confidence": 0.99},
    {"source": "pyobo", "prefix": "mesh", "confidence": 0.99},
    {"source": "pyobo", "prefix": "ncit", "confidence": 0.99},
    {"source": "pyobo", "prefix": "umls", "confidence": 0.99}
  ],
  "priority": ["uberon", "mesh", "bto", "caro", "ncit", "umls"],
  "mutations": [
    {"source": "uberon", "confidence": 0.8},
    {"source": "bto", "confidence": 0.65},
    {"source": "caro", "confidence": 0.8},
    {"source": "ncit", "confidence": 0.7},
    {"source": "umls", "confidence": 0.7}
  ],
  "subsets": {
    "mesh": ["mesh:D001829", ..., "mesh:D056224"],
    "ncit": ["ncit:C12219"],
    "umls": ["sty:T024", "sty:T017"]
  },
  "keep_prefixes": ["uberon", "mesh", "bto", "caro", "ncit", "umls"],
  "remove_imprecise": false,
  "processed_neo4j_name": "semra-anatomy",
  "add_labels": false
}
```

Supplementary Figure S2: The declarative configuration for the Anatomy domain. MeSH subsets truncated for clarity. Additional documentation on configurations at `https://semra.readthedocs.io/en/latest/pipeline.html`

The declarative configuration (**Supplementary Figure S2**) incorporates several fields:

1. *Name* a title for the configuration
2. *Inputs*, each containing a source API (e.g., PyOBO, Wikidata, Bioontologies, Custom), a prefix, and a consumer-provided confidence
3. *Subsets*, which allow for specifying when only an explicit list of sub-hierarchies in the resource should be used. This is only applicable for ontology-like resources
4. *Mutations*, a list of mutations objects. Commonly, this has a combination of a source and confidence, meaning that all database cross-references from the source can be implied to mean exact matches with the given confidence (besides many-to-many mappings, which are automatically removed)
5. *Keep Prefixes*, a list of prefixes to subset all mappings to before processing. *Post Keep Prefixes* does the same filter, but after processing. This is useful for incorporating resources like UMLS in the mapping process, but when it's not desirable to retain mappings with UMLS entities due to licensing questions
6. *Priority*, the priority order for selecting a canonical entity to represent a clique of equivalent entities
7. *Remove Imprecise*, a flag for removing database cross-references that couldn't be inferred as exact matches with adequate confidence
8. *Add Labels*, and other flags not shown, affect output by adding names to the SSSOM file. This can make processing take much longer as it requires caching all relevant external resources.
9. Not Shown: configuration for making outputs

Mapping landscapes for specific entity types

Resource	License	Version	Terms	Scope
BTO [8]	CC-BY-4.0	2021-10-26	6,570	F
CCLE [6]	ODbL-1.0	2019	1,061	F
Cellosaurus [2]	CC-BY-4.0	51.0	159,463	F
CL [5]	CC-BY-4.0	2025-02-13	3,046	F
CLO [12]	CC-BY-3.0	2.1.188	39,125	F
DepMap [3]	CC-BY-4.0	24Q4	1,814	F
EFO [10]	Apache-2.0	3.76.0	27	S
MeSH [11]	CC0-1.0	2025	636	S
NCIT [7]	CC-BY-4.0	25.03c	503	S
UMLS [4]	Custom	2024AB	6,312	S

Supplementary Table S1: Cell type and cell line landscape statistics showing the license for each resource, the version used to generate the results, the number of terms appearing in the landscape, and the scope for the inclusion of the resource: F is the full resource was used and S if a subset was used specific to cell types and cell lines.

Resource	License	Version	Terms	Scope
DO [13]	CC0-1.0	2025-03-03	14,220	F
EFO [10]	Apache-2.0	3.76.0	2,121	S
GARD [9]	Unknown		6,132	F
ICD10	Custom	2019	2,345	F
ICD10-CM	Custom		2,830	O
ICD11	CC-BY-ND	2025-01	71,175	F
ICD9	CC-BY-ND		3,955	O
ICD9-CM	CC-BY-ND		2,226	O
ICD-O	CC-BY-ND		797	O
MeSH [11]	CC0-1.0	2025	3,178	S
MONDO [14]	CC-BY-4.0	2025-03-04	29,826	F
NCIT [7]	CC-BY-4.0	25.03c	20,522	S
OMIM [1]	Custom	2025-03-24	14,452	O
OMIMPS [1]	Custom	2025-03-24	588	F
Orphanet [15]	CC-BY-4.0	4.6	15,507	F
UMLS [4]	Custom	2024AB	187,376	S

Supplementary Table S2: Disease landscape statistics, showing the license for each resource, the version used to generate the results, the number of terms appearing in the landscape, and the scope for the inclusion of the resource: F if the full resource was used, S if a subset specific to diseases, or O if only terms were included that were observed as mappings from other resources.

Code examples

In this supplementary section, we include several code examples for using the SeMRA Python package. Additional examples, tutorials, and reference documentation can be found on the README of the SeMRA GitHub repository (`https://github.com/biopragmatics/semra#readme`) and on the official documentation, hosted on ReadTheDocs at `https://semra.readthedocs.io`.

Data Model The first example demonstrates SeMRA's object, provenance, and cascading confidence model:

```python
from semra import *

r1 = Reference.from_curie("chebi:107635", name="2,3-diacetyloxybenzoic")
r2 = Reference.from_curie("mesh:C011748", name="tosiben")
```

```python
mapping = Mapping(
    subject=r1, predicate=EXACT_MATCH, object=r2,
    evidence=[
        SimpleEvidence(
            justification=MANUAL_MAPPING,
            confidence=0.99,
            author=Reference(
                prefix="orcid",
                identifier="0000-0003-4423-4370",
                name="Charles Tapley Hoyt",
            ),
            mapping_set=MappingSet(
                name="biomappings", license="CC0", confidence=0.90,
            ),
        )
    ]
)
```

I/O The second example demonstrates importing semantic mappings from a variety of sources:

```python
import semra

# load mappings from any standardized SSSOM file as a file path or URL,
# via `pandas.read_csv`
sssom_url = "https://w3id.org/biopragmatics/biomappings/sssom/biomappings.sssom.tsv"
mappings = semra.from_sssom(
    sssom_url, license="spdx:CC0-1.0", mapping_set_title="biomappings",
)

# alternatively, metadata can be passed via a file/URL
mappings_alt = semra.from_sssom(
    sssom_url,
    metadata="https://w3id.org/biopragmatics/biomappings/sssom/biomappings.sssom.yml"
)

# load mappings from the Gene Ontology (via OBO format)
go_mappings = semra.from_pyobo("go")

# load mappings from the Uber Anatomy Ontology (via OWL format)
uberon_mappings = semra.from_bioontologies("uberon")
```

Chaining and Inference SeMRA implements the chaining and inference rules described in the SSSOM specification[1]. The first rule is *inversions*[2]:

```python
from semra import Mapping, EXACT_MATCH, Reference
from semra.inference import infer_reversible

r1 = Reference(prefix="chebi", identifier="107635", name="2,3-diacetyloxybenzoic")
r2 = Reference(prefix="mesh", identifier="C011748", name="tosiben")

mapping = Mapping(subject=r1, predicate=EXACT_MATCH, object=r2)
```

[1] https://mapping-commons.github.io/sssom/chaining-rules
[2] https://mapping-commons.github.io/sssom/chaining-rules/#inverse-rules

```python
# includes the mesh -> exact match-> chebi mapping with full provenance
mappings = infer_reversible([mapping])
```

SSSOM's second chaining and inference rule is about *transitivity*[3]. This means some predicates apply over chains. SeMRA further implements configuration for two-length chains and could be extended to arbitrary chains.

```python
from semra import Reference, Mapping, EXACT_MATCH
from semra.inference import infer_chains

r1 = Reference.from_curie("mesh:C406527", name="R 115866")
r2 = Reference.from_curie("chebi:101854", name="talarozole")
r3 = Reference.from_curie("chembl.compound:CHEMBL459505", name="TALAROZOLE")

m1 = Mapping(subject=r1, predicate=EXACT_MATCH, object=r2)
m2 = Mapping(subject=r2, predicate=EXACT_MATCH, object=r3)

# infers r1 -> exact match -> r3
mappings = infer_chains([m1, m2])
```

SSSOM's third chaining and inference rule is *generalization*[4], which means that a more strict predicate can be relaxed to a less specific predicate, like `owl:equivalentTo` to `skos:exactMatch`.

```python
from semra import Reference, Mapping, EXACT_MATCH
from semra.inference import infer_generalizations

r1 = Reference.from_curie("chebi:101854", name="talarozole")
r2 = Reference.from_curie("chembl.compound:CHEMBL459505", name="TALAROZOLE")

m1 = Mapping(subject=r1, predicate=EXACT_MATCH, object=r2)

mappings = infer_generalizations([m1])
```

Cypher example queries

The locally deployable graph database and web application can be queried directly with the Cypher query language[5] in one of the following ways:

1. By connecting with a client via the `bolt` protocol on port 7687, which is exposed in the Dockerfile
2. By navigating to http://localhost:7474 in the web browser to use Neo4j's builtin graphical front-end, where you can type in Cypher queries and interact with the results.

We include a subset of the example queries listed on the SeMRA main documentation at `https://semra.readthedocs.io/en/latest/cypher.html` to highlight the ways mappings and evidences can be queried:

[3] https://mapping-commons.github.io/sssom/chaining-rules/#transitivity-rule
[4] https://mapping-commons.github.io/sssom/chaining-rules/#generalisation-rules
[5] https://neo4j.com/docs/cypher-manual/current/introduction/

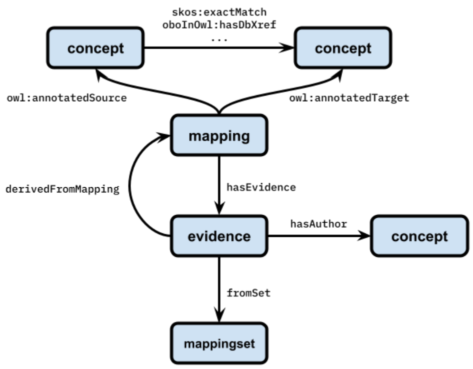

Supplementary Figure S3: A view over the SeMRA graph database schema.

Get all targets for exact match mappings where `cellosaurus:0440` is the source:

```
MATCH
    (source:concept)-[:`skos:exactMatch`]->(target:concept)
WHERE source.curie = "cellosaurus:0440"
RETURN target
```

The same query can be reified using `owl:annotatedSource`, `owl:annotatedTarget`, and the `mapping` node type:

```
MATCH
    (m:mapping)-[:`owl:annotatedSource`]->(source:concept) ,
    (m)-[:`owl:annotatedSource`]->(target:concept)
WHERE source.curie = "cellosaurus:0440" and m.predicate == "skos:exactMatch"
RETURN target
```

After reifying, you can extend the query to return evidences. In the interactive view, returning multiple elements will also automatically show edges between them:

```
MATCH
    (m:mapping)-[:`owl:annotatedSource`]->(source:concept) ,
    (m)-[:`owl:annotatedSource`]->(target:concept)
    (m)-[:hasEvidence]->(e:evidence)
WHERE source.curie = "cellosaurus:0440" and m.predicate == "skos:exactMatch"
RETURN source, target, m, e
```

Reification is useful for doing complex filters, e.g., on mapping justification. The following query returns exact matches to `cellosaurus:0440` that have manual mapping justification:

```
MATCH
    (m:mapping)-[:`owl:annotatedSource`]->(source:concept) ,
    (m)-[:`owl:annotatedSource`]->(target:concept)
    (m)-[:hasEvidence]->(e:evidence)
WHERE
    source.curie = "cellosaurus:0440"
    and m.predicate == "skos:exactMatch"
    and e.mapping_justification == "semapv:ManualMappingCuration"
RETURN target
```

The previous query can be reformulated to filter for minimum confidence:

```
MATCH
    (m:mapping)-[:`owl:annotatedSource`]->(source:concept) ,
    (m)-[:`owl:annotatedSource`]->(target:concept)
    (m)-[:hasEvidence]->(e:evidence)
WHERE
    source.curie = "cellosaurus:0440"
    and m.predicate == "skos:exactMatch"
    and e.confidence > 0.3
RETURN target
```

It can also be extended to return the authors of the evidences:

```
MATCH
    (m:mapping)-[:`owl:annotatedSource`]->(source:concept) ,
    (m)-[:`owl:annotatedSource`]->(target:concept)
    (m)-[:hasEvidence]->(e:evidence)
    (e)-[:hasAuthor]->(author:concept)
WHERE
    source.curie = "cellosaurus:0440"
    and m.predicate == "skos:exactMatch"
    and e.mapping_justification == "semapv:ManualMappingCuration"
RETURN target, author
```

The following query gets all mappings (with associated evidences, mapping sets, and authors) where `cellosaurus:0440` is the source, with optional matches for mapping sets and authors:

```
MATCH
    (m:mapping)-[:`owl:annotatedSource`]->(source:concept) ,
    (m:mapping)-[:`owl:annotatedTarget`]->(target:concept) ,
    (m)-[:hasEvidence]->(e:evidence)
WHERE source.curie = "cellosaurus:0440"
OPTIONAL MATCH
    (e)-[:fromSet]->(mset:mappingset)
OPTIONAL MATCH
    (e)-[:hasAuthor]->(author:concept)
RETURN source, target, m, e, mset, author
```

References

[1] J. S. Amberger, C. A. Bocchini, F. Schiettecatte, A. F. Scott, and A. Hamosh. Omim.org: Online mendelian inheritance in man (omim®), an online catalog of human genes and genetic disorders. *Nucleic Acids Research*, 43:D789–D798, 1 2015. ISSN 1362-4962. doi: 10.1093/nar/gku1205.

[2] A. Bairoch. The Cellosaurus, a cell-line knowledge resource. *Journal of biomolecular techniques : JBT*, 29: 25–38, 7 2018. ISSN 1943-4731 (Electronic). doi: 10.7171/jbt.18-2902-002.

[3] J. Barretina, G. Caponigro, N. Stransky, K. Venkatesan, A. A. Margolin, S. Kim, C. J. Wilson, J. Lehár, G. V. Kryukov, D. Sonkin, A. Reddy, M. Liu, L. Murray, M. F. Berger, J. E. Monahan, P. Morais, J. Meltzer, A. Korejwa, J. Jané-Valbuena, F. A. Mapa, J. Thibault, E. Bric-Furlong, P. Raman, A. Shipway, I. H. Engels, J. Cheng, G. K. Yu, J. Yu, P. Aspesi, M. de Silva, K. Jagtap, M. D. Jones, L. Wang, C. Hatton, E. Palescandolo, S. Gupta, S. Mahan, C. Sougnez, R. C. Onofrio, T. Liefeld, L. MacConaill, W. Winckler, M. Reich, N. Li, J. P. Mesirov, S. B. Gabriel, G. Getz, K. Ardlie, V. Chan, V. E. Myer, B. L. Weber, J. Porter, M. Warmuth, P. Finan, J. L. Harris, M. Meyerson, T. R. Golub, M. P. Morrissey, W. R. Sellers, R. Schlegel, and L. A. Garraway. The Cancer Cell Line Encyclopedia enables predictive modelling of anticancer drug sensitivity. *Nature*, 483(7391):603–607, 2012. ISSN 1476-4687. doi: 10.1038/nature11003. URL https://doi.org/10.1038/nature11003.

[4] O. Bodenreider. The unified medical language system (umls): integrating biomedical terminology. *Nucleic Acids Research*, 32:267D–270, 1 2004. ISSN 1362-4962. doi: 10.1093/nar/gkh061.

[5] A. D. Diehl, T. F. Meehan, Y. M. Bradford, M. H. Brush, W. M. Dahdul, D. S. Dougall, Y. He, D. Osumi-Sutherland, A. Ruttenberg, S. Sarntivijai, C. E. Van Slyke, N. A. Vasilevsky, M. A. Haendel, J. A. Blake, and C. J. Mungall. The Cell Ontology 2016: enhanced content, modularization, and ontology interoperability. *J. Biomed. Semantics*, 7(1):44, 2016. ISSN 2041-1480. doi: 10.1186/s13326-016-0088-7. URL `https://doi.org/10.1186/s13326-016-0088-7`.

[6] M. Ghandi, F. W. Huang, J. Jané-Valbuena, G. V. Kryukov, C. C. Lo, E. R. McDonald, J. Barretina, E. T. Gelfand, C. M. Bielski, H. Li, K. Hu, A. Y. Andreev-Drakhlin, J. Kim, J. M. Hess, B. J. Haas, F. Aguet, B. A. Weir, M. V. Rothberg, B. R. Paolella, M. S. Lawrence, R. Akbani, Y. Lu, H. L. Tiv, P. C. Gokhale, A. de Weck, A. A. Mansour, C. Oh, J. Shih, K. Hadi, Y. Rosen, J. Bistline, K. Venkatesan, A. Reddy, D. Sonkin, M. Liu, J. Lehar, J. M. Korn, D. A. Porter, M. D. Jones, J. Golji, G. Caponigro, J. E. Taylor, C. M. Dunning, A. L. Creech, A. C. Warren, J. M. McFarland, M. Zamanighomi, A. Kauffmann, N. Stransky, M. Imielinski, Y. E. Maruvka, A. D. Cherniack, A. Tsherniak, F. Vazquez, J. D. Jaffe, A. A. Lane, D. M. Weinstock, C. M. Johannessen, M. P. Morrissey, F. Stegmeier, R. Schlegel, W. C. Hahn, G. Getz, G. B. Mills, J. S. Boehm, T. R. Golub, L. A. Garraway, and W. R. Sellers. Next-generation characterization of the Cancer Cell Line Encyclopedia. *Nature*, 569(7757): 503–508, 2019. ISSN 1476-4687. doi: 10.1038/s41586-019-1186-3. URL `https://doi.org/10.1038/s41586-019-1186-3`.

[7] J. Golbeck, G. Fragoso, F. Hartel, J. Hendler, J. Oberthaler, and B. Parsia. The National Cancer Institute's thésaurus and ontology. *Journal of Web Semantics*, 1:75–80, 12 2003. ISSN 15708268. doi: 10.1016/j.websem.2003.07.007.

[8] M. Gremse, A. Chang, I. Schomburg, A. Grote, M. Scheer, C. Ebeling, and D. Schomburg. The BRENDA Tissue Ontology (BTO): the first all-integrating ontology of all organisms for enzyme sources. *Nucleic Acids Res.*, 39 (suppl_1):D507–D513, jan 2011. ISSN 0305-1048. doi: 10.1093/nar/gkq968. URL `https://doi.org/10.1093/nar/gkq968`.

[9] A. Hoskins. Genetic and rare diseases information center (gard). *Medical reference services quarterly*, 41: 389–394, 2022. ISSN 1540-9597. doi: 10.1080/02763869.2022.2131143.

[10] J. Malone, E. Holloway, T. Adamusiak, M. Kapushesky, J. Zheng, N. Kolesnikov, A. Zhukova, A. Brazma, and H. Parkinson. Modeling sample variables with an Experimental Factor Ontology. *Bioinformatics*, 26(8):1112–1118, 03 2010. ISSN 1367-4803. doi: 10.1093/bioinformatics/btq099. URL `https://doi.org/10.1093/bioinformatics/btq099`.

[11] F. B. Rogers. Medical subject headings. *Bull. Med. Libr. Assoc.*, 51:114–6, jan 1963. ISSN 0025-7338. URL `http://www.ncbi.nlm.nih.gov/pubmed/13982385http://www.pubmedcentral.nih.gov/articlerender.fcgi?artid=PMC197951`.

[12] S. Sarntivijai, Y. Lin, Z. Xiang, T. F. Meehan, A. D. Diehl, U. D. Vempati, S. C. Schürer, C. Pang, J. Malone, H. Parkinson, Y. Liu, T. Takatsuki, K. Saijo, H. Masuya, Y. Nakamura, M. H. Brush, M. A. Haendel, J. Zheng, C. J. Stoeckert, B. Peters, C. J. Mungall, T. E. Carey, D. J. States, B. D. Athey, and Y. He. CLO: The cell line ontology. *J. Biomed. Semantics*, 5(1):37, 2014. ISSN 2041-1480. doi: 10.1186/2041-1480-5-37. URL `https://doi.org/10.1186/2041-1480-5-37`.

[13] L. M. Schriml, J. B. Munro, M. Schor, D. Olley, C. McCracken, V. Felix, J. A. Baron, R. Jackson, S. M. Bello, C. Bearer, R. Lichenstein, K. Bisordi, N. C. Dialo, M. Giglio, and C. Greene. The Human Disease Ontology 2022 update. *Nucleic Acids Research*, 50(D1):D1255–D1261, 11 2021. ISSN 0305-1048. doi: 10.1093/nar/gkab1063. URL `https://doi.org/10.1093/nar/gkab1063`.

[14] N. A. Vasilevsky, N. A. Matentzoglu, S. Toro, J. E. Flack, H. Hegde, D. R. Unni, G. F. Alyea, J. S. Amberger, L. Babb, J. P. Balhoff, T. I. Bingaman, G. A. Burns, O. J. Buske, T. J. Callahan, L. C. Carmody, P. C. Cordo, L. E. Chan, G. S. Chang, S. L. Christiaens, L. C. Daugherty, M. Dumontier, L. E. Failla, M. J. Flowers, H. A. Garrett, J. L. Goldstein, D. Gration, T. Groza, M. Hanauer, N. L. Harris, J. A. Hilton, D. S. Himmelstein, C. T. Hoyt, M. S. Kane, S. Köhler, D. Lagorce, A. Lai, M. Larralde, A. Lock, I. L. Santiago, D. R. Maglott, A. J.

Malheiro, B. H. M. Meldal, M. C. Munoz-Torres, T. H. Nelson, F. W. Nicholas, D. Ochoa, D. P. Olson, T. I. Oprea, D. Osumi-Sutherland, H. Parkinson, Z. M. Pendlington, A. Rath, H. L. Rehm, L. Remennik, E. R. Riggs, P. Roncaglia, J. E. Ross, M. F. Shadbolt, K. A. Shefchek, M. N. Similuk, N. Sioutos, D. Smedley, R. Sparks, R. Stefancsik, R. Stephan, A. L. Storm, D. Stupp, G. S. Stupp, J. C. Sundaramurthi, I. Tammen, D. Tay, C. L. Thaxton, E. Valasek, J. Valls-Margarit, A. H. Wagner, D. Welter, P. L. Whetzel, L. L. Whiteman, V. Wood, C. H. Xu, A. Zankl, X. A. Zhang, C. G. Chute, P. N. Robinson, C. J. Mungall, A. Hamosh, and M. A. Haendel. Mondo: Unifying diseases for the world, by the world. *medRxiv*, 2022. doi: 10.1101/2022.04.13.22273750. URL `https://www.medrxiv.org/content/early/2022/05/03/2022.04.13.22273750`.

[15] S. S. Weinreich, R. Mangon, J. J. Sikkens, M. E. en Teeuw, and M. C. Cornel. [orphanet: a european database for rare diseases]. *Nederlands tijdschrift voor geneeskunde*, 152:518–9, 3 2008. ISSN 0028-2162.
